# Photodynamic Detection of Peritoneal Metastases Using 5-Aminolevulinic Acid (ALA)

**DOI:** 10.3390/cancers9030023

**Published:** 2017-03-01

**Authors:** Yutaka Yonemura, Yoshio Endo, Emel Canbay, Yang Liu, Haruaki Ishibashi, Akiyoshi Mizumoto, Masamitu Hirano, Yuuki Imazato, Nobuyuki Takao, Masumi Ichinose, Kousuke Noguchi, Yan Li, Satoshi Wakama, Kazuhiro Yamada, Koutarou Hatano, Hiroshi Shintani, Hiroyuki Yoshitake, Shun-ichiro Ogura

**Affiliations:** 1 NPO Organization to support Peritoneal Surface Malignancy Treatment, 510 Fukushima-Cho, Shimogyou-Ku, Kyoto 600-8189, Japan; 2 Peritoneal Surface Malignancy Center, Kishiwada Tokushukai Hospital, 4-27-1 Kamori-Cho, Kishiwada City, Osaka 596-8522, Japan; LYMIKELEO@hotmail.com (Y.L.); akebono_jaguar@yahoo.co.jp (S.W.); iceman0710@hotmail.co.jp (K.Y.); ko-ta-music@kzd.biglobe.ne.jp (K.H.); hiroshihiroshi.s@gmail.com (H.S.); tennis.ce.surge@gmail.com (H.Y.); 3 Department of General Surgery, Kusatsu General Hospital, 1660 Yabase, Kusatsu City, Shiga 525-8585, Japan; mizumotoakiyoshi1206@yahoo.co.jp (A.M.); hirano@kusatsu-gh.or.jp (M.H.); omokage.0117@gmail.com (Y.I.); s380402@yahoo.co.jp (N.T.); michi@yd6.so-net.ne.jp (M.I.); nogupinbad@yahoo.co.jp (K.N.); 4 Central Research Resource Center, Cancer Research Institute, Kanazawa University, Kakuma-machi, Kanazawa 920-1192, Japan; yendo2@staff.kanazawa-u.ac.jp; 5 NPO HIPEC Istanbul, Guzelbahce Sokak No:15 Nisantasi, Istanbul 34367, Turkey; drecanbay@gmail.com; 6 Department of Peritoneal Surface Oncology, Beijing Shijitan Hospital of Capital Medical University, Beijing 100038, China; liyansd2@163.com; 7 Graduate School of Bioscience and Biotechnology, Tokyo Institute of Technology, 4259 Nagatsu-Cho, Midori-ku, Yokohama 226-8501, Japan; sogura@bio.titech.ac.jp

**Keywords:** aminolevulinic acid, photodynamic diagnosis (PDD), peritoneal surface malignancies, PEPT1, ABCG2, ferrochelatase

## Abstract

In the past, peritoneal metastasis (PM) was considered as a terminal stage of cancer. From the early 1990s, however, a new comprehensive treatment consisting of cytoreductive surgery and perioperative chemotherapy has been established to improve long-term survival for selected patients with PM. Among prognostic indicators after the treatment, completeness of cytoreduction is the most independent predictors of survival. However, peritoneal recurrence is a main cause of recurrence, even after complete cytoreduction. As a cause of peritoneal recurrence, small PM may be overlooked at the time of cytoreductive surgery (CRS), therefore, development of a new method to detect small PM is desired. Recently, photodynamic diagnosis (PDD) was developed for detection of PM. The objectives of this review were to evaluate whether PDD using 5-aminolevulinic acid (ALA) could improve detection of small PM.

## 1. Introduction

In the past, peritoneal metastasis (PM) was considered as a terminal stage of cancer, and patients were treated with palliative surgery or chemotherapy. From the early 1990s a new comprehensive treatment consisting of cytoreductive surgery (CRS) and perioperativeintraperitoneal and systemic chemotherapy has been considered as an effective treatment modality that can provide long-term survival for select patients with PM [1,2,3,4]. Among prognostic indicators after comprehensive treatment, completeness of cytoreduction and extent of disease are the most independent predictors of survival. However, peritoneal recurrence develops in about 70% of patients, even after complete cytoreduction [5,6]. As a cause of peritoneal recurrence after complete resection, small PM may be overlooked at the time of CRS. Therefore, the development of a new method to detect small PM is desired. Recently, photodynamic diagnosis (PDD) using aminolevulinic acid was developed for detection of PM from gastrointestinal cancer, ovarian cancer, and mesothelioma. The present review represents recent results of PDD to detect PM. The literature in PubMedwas searched in June 2016 by combining synonyms of “PDD”, cancer and peritoneal metastasis.

## 2. Rationale of Photodynamic Diagnosis (PDD) to Detect Peritoneal Metastasis Using 5-Amino-levulinic Acid

**Figure 1:**
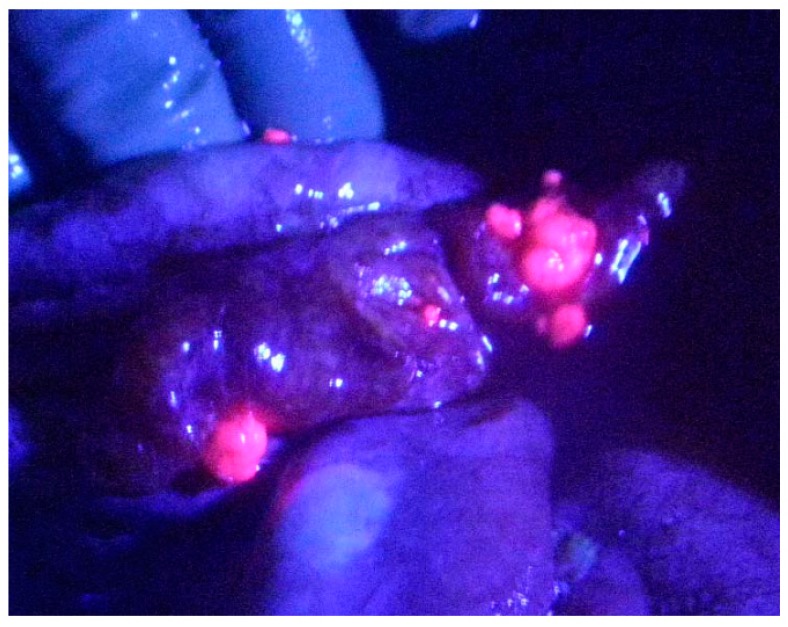
Peritoneal metastasis from ovarian cancer emitted strong red fluorescence under irradiation of violet light after oral administration of 5-aminolevulinic acid. Peritoneal metastases were emitted as red color.

**Figure 2:**
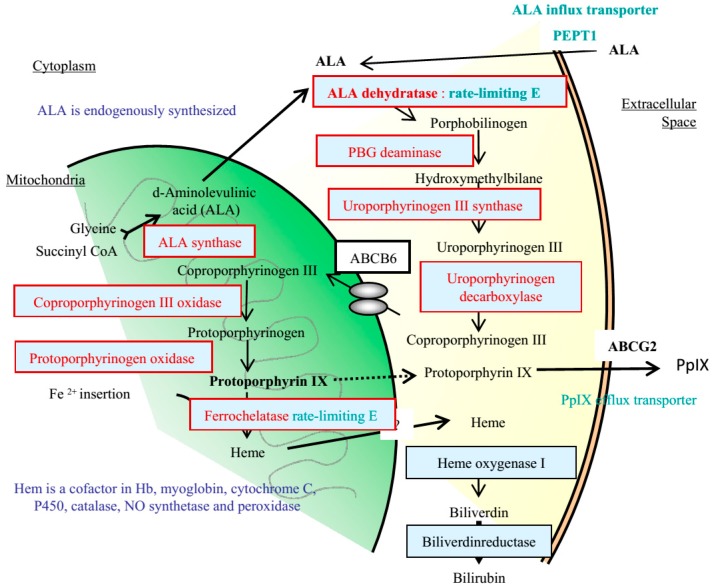
Biosynthesis pathway of PpIX and heme.

**Figure 3:**
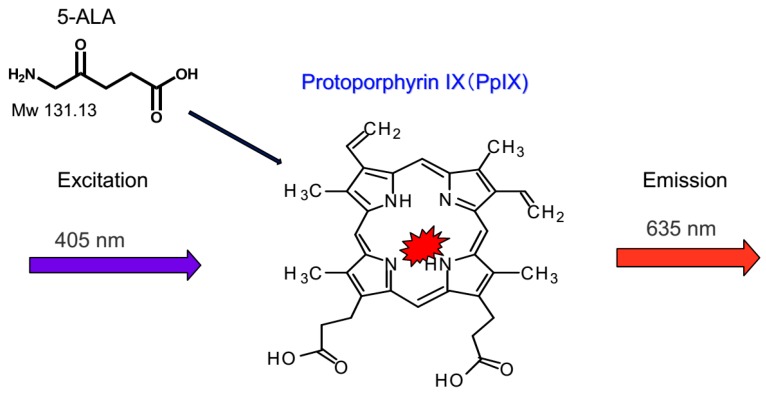
Molecular structure of 5-ALA, and 5-ALA-based PDD. Following excitation with blue light (λ = 400–410 nm), the PpIX emits a red-violet light of 635 nm. This phenomenon is potentially exploitable to detect tumor and is named 5-ALA fluorescence-guided surgery.

Initially, PDD for peritoneal malignancy had been proposed in animal models [7]. In those studies the identification of small PM was significantly increased by 5-aminolevulinic acid (ALA) administration followed by fluorescence detection (Figure 1) [8]. ALA is the natural precursor of protoporphyrin (Pp)IX and heme. Intrinsic ALA is synthesized from succinyl-CoA and glycine by ALA synthase, and ALA synthase is controlled by heme through a feedback mechanism. In the heme synthesis pathway, ALA is converted to porphobilinogen by ALA dehydratase as a rate-limiting enzyme, and metabolized to PpIX by 6 processes (Figure 2). After excess administration of ALA, ALA is accumulated in cancer cells through ALA influx transporter, expressed on cancer cell membrane. As a result, intracellular PpIX synthesis increases, and PpIX accumulates in cancer cells. PpIX in cancer cells emits a red fluorescence under violet light at 405 nm (Figure 3) [9,10,11].

Yonemura et al. reported that PpIX contents (0.0098 ± 0.0081 nm/mg-protein) of ALA-positive PM were significantly higher than those of ALA-negative PM (0.0019±0.0015 nm/mg-protein) (*p* = 0.0095) [12]. (I changed the reference)

## 3. Molecular Mechanisms of Selective Accumulation of 5-ALA and PpIX in Cancer Cells and Cancer Tissues

The reasons why PpIX excessively accumulates in cancer cells remain unclear. The following two hypotheses have been proposed. (1) PpIX accumulates due to decreased ferrochelatase activity in cancer cells [10]; (2) ALA has a high affinity for the malignant cells [11].

As shown in Figure 2, 8 enzymes contribute in the heme synthesis pathway, and up-regulation or down-regulation of these enzymes may influence the ALA fluorescence. In 5 gastric cancer cell lines, Hagiya et al. reported that expression level of these 8 enzymes were same and did not correlated with ALA fluorescence [13]. In the malignant glioma cells, the ferrochelatase activity was lower than in normal brain tissue [10]. In the normal brain cells, a large amount of PpIX was biosynthesized in the mitochondria after administration of ALA, but an excessive amount of PpIX metabolized into heme by ferrochelatase, resulting in the decrease of the PpIX contents. In contrast, PpIX accumulated in malignant glioma cells, because of lower content of ferrochelatase. In bladder cancer, ferrochelatase expression was downregulated, and the PpIX contents of tumor tissue increased after ALA administration [13].

Different from malignant glioma and bladder cancer, Yonemura et al. reported no correlation between ferrochelatase expression and ALA PDD fluorescence status in PM tissues [12]. Additionally, they reported that no relation was found between ferrochelatase expression and PpIX contents in PM tissues [12]. Accordingly, PpIX contents in PM did not depend on the ferrochelatase activities.

**Figure 4:**
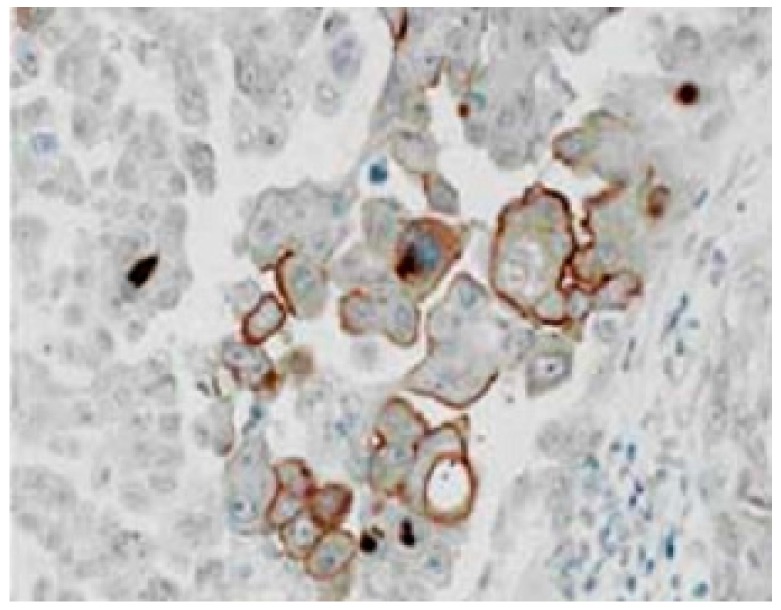
Immunohistologic finding of PEPT1 in peritoneal metastasis, using anti-PEPT1 rabbit polyclonal antibody H-235 (1:200 dilution; Santa Cruz Biotech, Santa Cruz, CA, USA).

Recently, the expressions of transporters of ALA and its metabolites were reported to contribute to the accumulation of PpIX [14,15]. ALA influx transporter (peptide transporter PEPT1) and porphyrin efflux transporter (ATP-binding cassette (ABC) transporter ABCG2) have important roles in regulating intracellular PpIX levels [11,13,16]. Our experimental study using gastric cancer cell lines suggested that upregulation of PEPT1 (ALA influx transporter) and downregulation of ABCG2 (porphyrin efflux transporter) genes could play pivotal roles in ALA-induced tumor specific PpIX accumulation [16]. PEPT1 is reportedly involved in the cellular uptake of ALA coupled with the co-transporter of H^+^/H_3_O^+^ [16,17]. PEPT1 immunoreactivity was found on the cancer cell membrane (Figure 4). Yonemura et al. reported that a significant increase of PpIX contents was found in PM showing up-regulation of PEPT1 expression [12]. Additionally, a significant correlation between PEPT1 mRNA expression and ALA PDD fluorescence status was found in clinical samples from PM [12,18].

**Figure 5:**
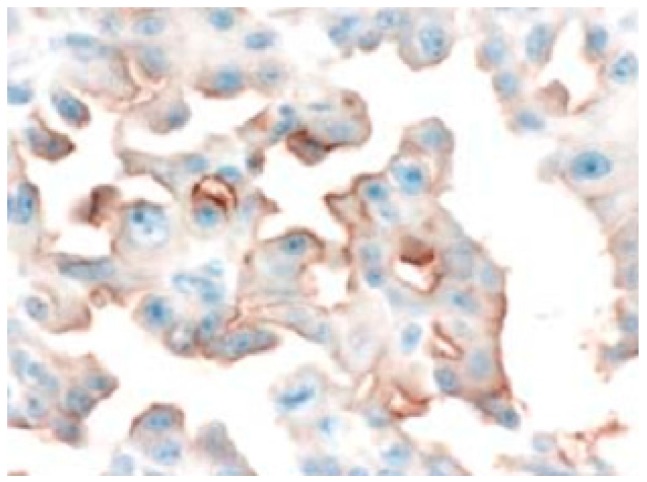
Immunohistologic finding of ABCG2 in peritoneal metastasis, using anti-ABCG2 mouse monoclonal antibody BXP-21 (1:200 dilution; Convance Research 152 Products, Emeryville, CA, USA). ABCG2 expression is detected on the cell membrane.

ABCG2 is known as an PpIX efflux transporter [14,15]. ABCG2 is expressed on the cell membrane of cancer cells (Figure 5). A significant correlation of ABCG2 mRNA expression and ALA PDD fluorescence status was reported [12]. Yonemura et al. also reported a significant correlation between PEPT1 mRNA and ABCG2 mRNA expression in PM tissues [12]. In PM with simultaneous expression of ABCG2 and PEPT1 mRNA, 88.2% (15/17) of PM showed positive fluorescence. In PM with upregulation of ABCG2 and downregulation of PEPT1 gene, fluorescence was not detected in 75% (6/8) [12].

As shown in Figure 2, ABCB6 has a role to transport coproporphyrinogen III from cytoplasm to mitochondria. If ABCB6 is up-regulated, ALA fluorescence may be enhanced. In clinical sampls of bladder cancer showing ALA fluorescence, however, ABCB6 was downregulated [13]. In contrast, expression of ABCB6 did not correlated with ALA fluorescence in gastric cancer cell lines [16]. Accordingly, expression of ABCB6 may not have an important role role in ALA fluorescence.

**Figure 6:**
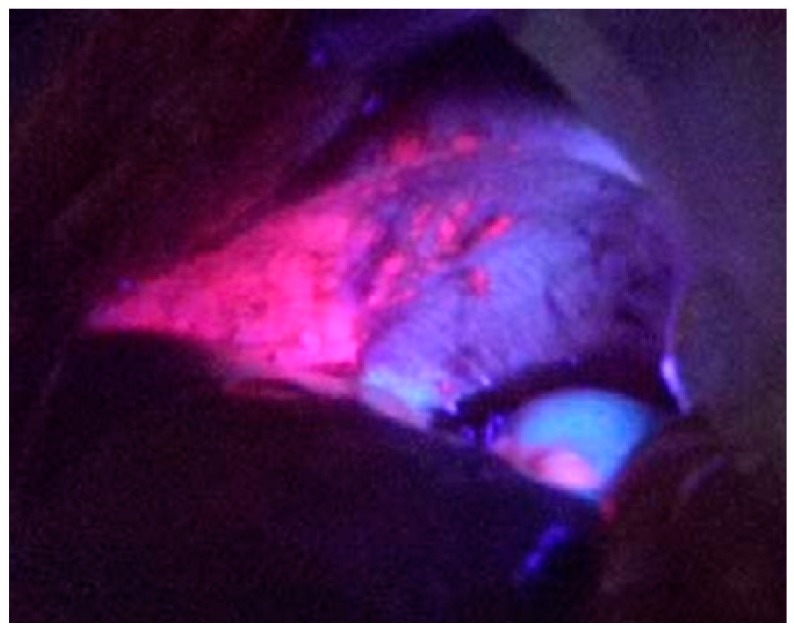
Intraoperative finding of PDD in a patient with peritoneal metastasis from appendiceal mucinous neoplasm on right subdiaphragmatic peritoneum. Peritoneal metastasis with mucinous materials were emitted with red fluorescence by violet right.

**Figure 7:**
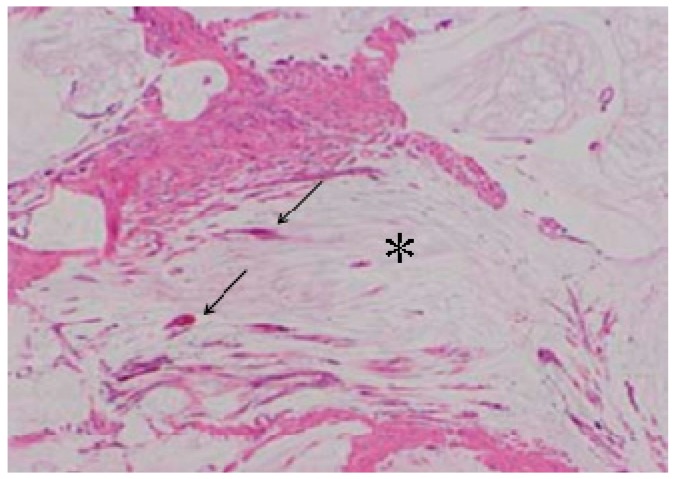
Histologic finding of peritoneal metastasis from appendiceal mucinous neoplasm (Figure 7). Mucinous materials (*) produced from cancer cells (→) extensively occupied stromal tissue.

After administration of an excess amount of ALA, ALA is transported into cancer cells in PM through PEPT1. The heme synthesis pathway is then activated by ALA dehydratase, which is a rate-limiting enzyme, and the intracellular PpIX contents increase (Figure 2). Yonemura et al. reported that most of PM with PEPT1 overexpression upregulated ferrochelatase and ABCG2 mRNA [12,18,19]. Accordingly, a part of PpIX is rapidly metabolized to heme by ferrochelatase. At the same time, PpIX is excreted from cancer cells into the stromal tissue through ABCG2 (PpIX efflux transporter), resulting in the accumulation of excess PpIX in the stromal tissue in the vicinity of cancer cells. The typical finding was observed in a case of appendiceal mucinous neoplasm expressing ABCG2 and PEPT1 (Figure 6). Cancer cells in PM tissue of appendiceal mucinous neoplasm are scarce, and mucinous materials produced from cancer cells extensively occupies the stromal tissue. PpIX accumulated in the mucinous material (Figure 6 and Figure 7) emitted strong red fluorescence by PDD.

In bladder cancer [13] and gastric cancer cell lines [16], ALA fluorescence status closely correlates with up-regulation of PEPT1 and down-regulation of ABCG2 gene. Accordingly, PDD status of these tumors depends on the expression of ferrochelatase. In PM tissues, however, positive ALA fluorescence depends on the simultaneous up-regulation of PEPT1 and ABCG2 gene, which increase PpIX contents in PM tissues.

Normal peritoneal tissue does not show photoemission under violet light at ALA PDD. Yonemura et al. reported that PpIX contents in the normal peritoneal tissue (0.0073 ± 0.0041 nm/mg/protein) were significantly lower than those in PM tissues (0.0109 ± 0.0031 nm/mg/protein) [12]. In the normal peritoneal tissues PEPT1, ABCG2 and ferrochelatase mRNA are expressed in 25.0% (10/40), 48.4% (18/40) and 70.0% (28/40) [20]. In normal mesothelial cells, PpIXmay be excreted into the peritoneal cavity by ABCG2 transporter and is metabolized to heme by ferrochelatase, resulting in the low PpIX contents [12]. Accordingly, the PpIX contents in normal peritoneal tissues are not sufficient to emit red fluorescence by violet light.

### 3.1. Immuhohistological Expressions of PEPT1 and ABCG2 Protein

Table 1 shows the correlation between PEPT1 immunoreactivity and ALA fluorescence status in 75 clinical samples of PM. PEPT1 immunoreactivity was significantly associated with ALA fluorescence status (*p* = 0.0019). Additionally, ALA fluorescence status was significantly related to ABCG2 immunoreactivity (Table 2, *p* = 0.0048) [12]. These results indicate that patients who are indicated for ALA PDD can be selected by PEPT1 and ABCG2 tissue status using immuno-histochemistry [12].

However, some ALA-positive PM express neither PEPT1 nor ABCG2, and ALA-negative PM showed simultaneous expression of PEPT1 and ABCG2. Little is known about how the PpIX crosses the mitochondria membrane back into the cytoplasm. Other unknown ALA influx or porphyrin efflux transporters may associate with accumulation of PpIX. In addition, changes in Fe^2+^ availability and environmental factors such as pH, blood supply and lymphatic circulation may be responsible for the selective accumulation of PpIX in PM tissues.

### 3.2. ALA PDD in Experimental PM

Several experimental studies investigating PDD for PM were published [21,22,23,24]. In experimental studies, ALA was administered intravenously or intraperitoneally. Hornung et al. [23] reported an experimental study of ALA PDD using PM induced in Fischer 344 rats by intraperitoneal injection of ovarian cancer cells. Four weeks after intraperitoneal inoculation, ALA 100 mg/kg was injected intravenously, and diagnostic laparotomy was performed at 1, 3, 6, 9 h thereafter. One to 3 h after IV injection is optimal for PDD [23].

Canis et al. injected 100 mg/kg of ALA in the peritoneal cavity of rats with PM from ovarian cancer cell line [21]. PDD was performed using endoscopy 3 h after ALA administration. Gahlen et al. injected 440 to 550 mg/kg of ALA intraperitoneally, and PDD was performed 4 h after injection [22].

Lüdicke et al. [23] reported the feasibility of detecting micrometastases in an ovarian cancer animal model using intraperitoneal administration of hexaminolaevulinate. Tumor-free peritoneum did not show fluorescence and was distinguishable from cancer nodules. The number of PM detected by PDD blue light mode was significantly higher than when using standard white light inspection twice as many more cancer lesions were detected by fluorescence than by white light inspection [21,22,23]. Experimental studies were able to detect occult PM of 0.1 mm to 0.4 mm in diameter [21,22]. By meticulous histological study, there was no false-positive findings in ALA fluorescence positive PM [25]. Gahlen et al. compared the effectiveness of PDD for experimental PM between intraperitoneal and intravenous injection of ALA [22]. They concluded that fluorescence laparoscopy after intraperitoneal photosensitization with ALA was a more reliable and effective method than systemic photosensitization for the detection of small or occult PM [22].

## 4. Clinical Application of ALA PDD to Detect Peritoneal Metastasis: ALA-Guided Cytoreductive Surgery

### 4.1. Methods of ALA PDD and ALA-Guided Cytoreductive Surgery

Before application of ALA, porphyria should be excluded in all patients anamnestically and by Hoesch test [25]. If patients have a history of porphyrias, ALA PDD should be cancelled. For ALA PDD, there are three administration routes of ALA, which are intraperitoneal (IP), intravenous (IV) and oral administration.

Intraperitoneal administration of ALA may be useful, because higher dose intensity of ALA in the peritoneal cavity can be obtained as compared with IV or oral administration. In Ip administration of ALA, surgeons should wait several hours after IP administration of ALA into the abdominal cavity by laparoscopy before starting ALA PDD [20]. ALA may not penetrate into deep seated cancer cells. However, ALA is absorbed from peritoneal lymphatics and blood vessels, and then reaches PM tissue from the blood circulation [20]. Accordingly, ALA PDD was recommended to start 3 to 5 h after IP administration [20,26]. Higher dose of ALA can be administered intraperitoneally as compared with IV administration, because high dose IV administration of ALA may cause side effects.

ALA can be administered definitely by intravenous application. Hormung et al. reported an experimental study after intravenous administration of ALA to detect PM [23]. Maximum fluorescence in PM was found on 1 to 3 h after IV infusion of 100 mg/kg of ALA. Gahlen et al. reported that fluorescence laparoscopy after IP photosensitization with ALA was a more reliable and effective method than IV photosensitization for the detection of small or occult PM [22]. As compared with IP administration, oral application is convenient and non-invasive [18,26].

In oral application of ALA, patients received 20mg/kg body weight of 5-ALA (Cosmo Bio Co., Ltd., Tokyo, Japan) dissolved in 50–100 mL of orange juice. The mixture was given orally 2 h before surgery. After oral administration, patients were kept away from direct sunlight for 24 h. After laparotomy, standard evaluation of the distribution and size of PM was conducted under white light, and peritoneal cancer index was calculated [27,28]. Tumor tissues and normal peritoneum were discriminated under white light. All lights in the operation room were then turned off. PDD was performed using a xenon lamp (300W) with violet light of a wavelength range of 375–445 nm for fluorescence excitation, and all peritoneal sectors were observed [27].

**Figure 8:**
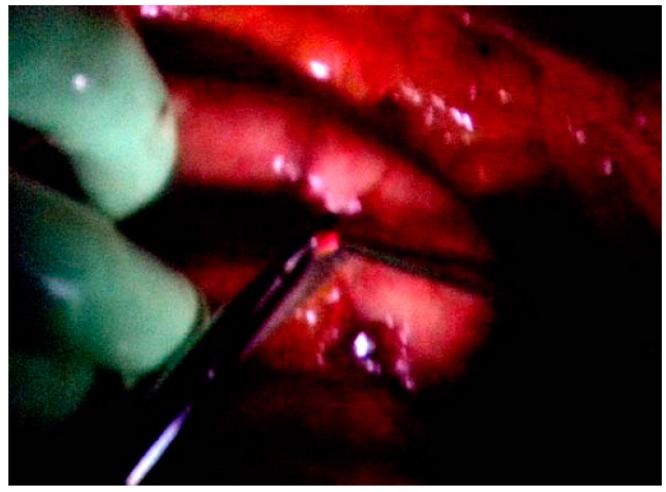
ALA guided cytoreductive surgery. Residual small nodules are removed under ALA PDD after macroscopic complete resection of PM under white light.

Cytoreductive surgery (CRS) and hyperthermic intraperitoneal chemotherapy were then performed [1]. After complete removal of macroscopic tumor, the residual tumors were searched using ALA PDD again (Figure 8).

### 4.2. Results of ALA PDD for Detection of Peritoneal Metastases from Various Cancers

Experimental and clinical studies demonstrated that ALA PDD was able to detect PM from different cancers [8,9,29,30]. In the study of ovarian cancer, Löning et al. reported that ALA-positive PM was detected in 12 (92.3%) of 13 patients [20]. In gastric cancer, the accuracy of the fluorescence imaging by laparoscopy was higher than that of white light imaging [29]. Watanabe et al. reported that the false-positive rate by white light imaging was 14.2% (2/14), but no false-positive or false-negative results were experienced under fluorescence imaging [29]. Liu et al. reported that the specificity of ALA PDD in 40 tumor specimens was 100%, and the false-negative rate was 7.5% in 40 non-fluorescence areas [8]. Yonemura et al. described promising results in detection of PM with ALA PDD [12,18]. Even though the overall detection rate was 56.6% (81/143), PDD enabled enhanced detection of metastatic nodules in the majority of ovarian cancer (84.6%), mesothelioma (62.5%) and pancreatic cancer (75.0%) specimens (Table 3) [12]. In addition, metastatic tumor nodules could be detected in 60.0% of PM from colorectal cancer [12]. In PM from gastric cancer and appendiceal mucinous neoplasms, however, the detection rates by PDD were low, at 25.7% and 16.4% (Table 3), respectively. ALA fluorescence depends on the tumor-specific accumulation of photosensitizing PpIX after the administration of ALA. As shown in Table 4, the PpIX contents in PM from gastric cancer and appendiceal mucinous carcinomas were significantly lower than those from ovarian cancer, pancreas cancer and mesothelioma [12].

Liu et al. reported that ALA PDD could detect a small tumor with diameter of 0.5 mm [8]. Accordingly, ALA-PDD is an accurate and reliable method to detect small PM.

As shown in Table 3, PM in 62 (43.4%) of 143 cases did not show positive ALA-fluorescence. False-negative results in ALA PDD were reported in the brain tumors and ovarian cancer [12,19].

Table 5 shows the results of four clinical studies of ALA PDD to detect PM. ALA was administered orally in four and intraperitoneally in one study. Dose of ALA ranged from 10 to 30 mg/kg. These results indicate the optimal dose of ALA for oral administration is 20 mg/kg. Incubation time after intraperitoneal administration was 5 h, but was 2 to 3 h after oral administration. Sensitivity ranged from 46% to 100%, and specificity was 100% in 4 reports. Loning et al. reported that endometriosis showed ALA fluorescence [20].

ALA fluorescence did not correlate with histo-pathological subtype, histological grading or amount of stroma [12]. ALA fluorescence depends on the amount of photosensitizing PpIX in PM tissues, location of tumor cells from peritoneal surface, and the preoperative chemotherapy. Cancer cells locate in the deep subperitoneal tissue cannot be detected by PDD [19]. Degenerated PM after preoperative chemotherapy may decrease ALA fluorescence intensity [12].

## 5. Application for Fertility Sparing Surgery

Fertility sparing surgery (FSS) is defined as surgery in which the uterus and ovaries are preserved for young women who desire childbearing [31]. FSS is now indicated for PM from ovarian cancer in stage 1, pseudomyxoma peritonei, or mesothelioma with low PCI [31] and FSS is indicated for patients younger than 41 years old. In FSS, one or both ovaries that are not involved by metastasis are preserved [31]. Biopsy from preserved ovaries may cause adhesion, and adhesion may cause sterility. Accordingly, it is very difficult to diagnose macroscopically whether an ovary is involved or not.

**Figure 9:**
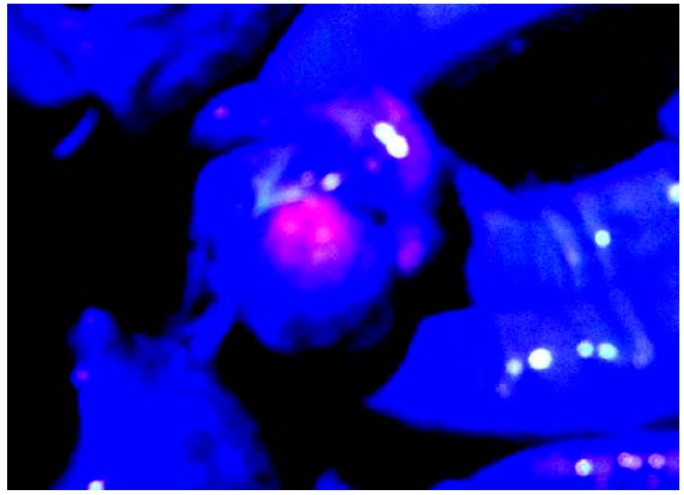
Ovarium of 35 years old woman with peritoneal metastasis from colon cancer is emitted by ALA PDD.

**Figure 10:**
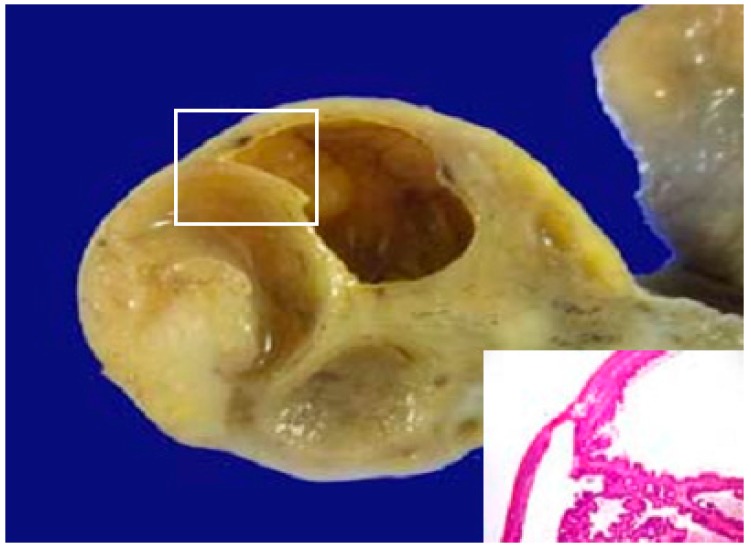
Histological examination revealed metastasis in ovary, which was emitted by ALA PDD.

From these circumstances, ALA PDD to detect ovarian metastasis was started [19], because ALA PDD is easy to perform and not invasive to ovarian tissue. Figure 9 shows an intraoperative photograph of ovarium of a 35-year-old woman with PM from colon cancer. Red fluorescence is observed in the ovarium by ALA PDD. Histological examination revealed metastasis in ovary (Figure 10). ALA PDD may be an effective and non-invasive method to determine involved ovary that should be removed.

## 6. Safety and Feasibility of ALA PDD

Side effects after oral administration of ALA were very few, because ALA is an intrinsic molecule and is rapidly metabolized to PpIX and heme through porphyrin/heme pathway. No permanent side effects of ALA was reported [8,12]. Yonemura et al. reported that nausea and vomiting occurred in only one (0.7%) of 138 patients who received oral administration of 20 mg/kg. of ALA for PDD [12]. Kamp et al. reported that postoperative serum liver enzymes and leucocytes were not significantly changed as compared to the preoperative values [32]. Kamp et al. reported that five (6.0%) of 84 patients suffered from transient erythema after unintentional exposure to daylight [32]. Porphyrias are a group of inherited or acquired metabolic disorders resulting from a deficiency in one of the eight enzymes involved in the biosynthesis of heme (Figure 2) [33,34]. Clinically, porphyrias that cause mainly neurological symptoms are classified as acute porphyrias, whereas those cause mainly skin photosensitivity are classified as cutaneous porphyria. Cutaneous porphyrias present with various types of skin symptoms due to phototoxicity associated with light exposure. During ALA PDD, care should be taken to avoid exposure to sunlight. Porphyrias are diagnosed by the detection of porphyrin-related metabolites in the urine, blood, or plasma. In some cases, diagnosis requires measurement of enzyme activity and gene studies [35]. If patients are diagnosed as acute porphyrias, treatment by drip infusion of large amount of glucose should be started. Intravenous administration of hematin or hemearginate has been reported to be effective in improving clinical symptoms and abnormal porphyrin metabolism [36]. Furthermore, Yonemura et al. reported that incidence of postoperative complications after ALA guided cytoreductive surgery was similar to that after non-ALA guided CRS [12].

## 7. Correlation between ALA PDD Status and Recurrence after CRS

Kamp et al. analyzed the correlation between local recurrence rate and the ALA induced fluorescent status in 84 patients who underwent ALA guided surgery for cerebral metastasis [32]. After surgical resection of cerebral metastasis, absence of 5-ALA-induced fluorescence may be a risk factor for local in-brain progression [32]. Possible explanation for the results is that ALA positive metastases were more radically resected than ALA negative metastasis. The surrounding tumor-free tissues of metastasis partially show ALA derived fluorescence, which might lead to unintended more radical resection [32].

In ALA positive PM, ALA PDD can increase the percentage of complete resection, and the rate of peritoneal recurrence could be decreased, when compared to conventional white-light surgery. However, no study about the relation between ALA fluorescent status and peritoneal recurrence after CRS for PM has been reported.

## 8. Conclusions

PDD is safe and feasible for detection of small PM from ovarian, pancreas, biliary, small bowel, colorectal cancer and mesothelioma. Small PM nodules which are overlooked under white light can be detected by ALA PDD. Simultaneous expression of PEPT1 and ABCG2 genes could have a pivotal role in PpIX accumulation in cancer tissues. Accordingly, preoperative examination for the expressions of PEPT1 and ABCG2 by immunohistological staining or reverse transcriptase-polymerase chain reaction may be useful for the selection of patients for ALA-PDD.

## Figures and Tables

**Table 1:** Correlation between PEPT1 immunoreactivity and PDD in PM (*p* = 0.0019) [20].

PEPT1 Expression	PDD Negative	PDD Positive	Total
Negative	22	10	32
Positive	14	29	43
Total	36	39	75

**Table 2:** Correlation between ABCG2 immunoreactivity and PDD in PM (*p* = 0.0048) [20].

ABCG2 mRNA Expression	PDD Negative	PDD Positive	Total
Negative	15	5	20
Positive	21	34	55
Total	36	39	75

**Table 3:** Positive emission rates by ALA PDD [12,19]. N = number of patients.

Primary Sites	Positive Emission Rates
Ovarian cancer *N* = 26	22/26 (84.6%)
Mesothelioma *N* = 8	5/8 (62.5%)
Pancreas cancer *N* = 4	3/4 (75%)
Colorectal cancer *N* = 29	27/45 (60%)
Biiary cancer *N* = 3	2/3 (66.7%)
Small bowel cancer *N* = 8	4/8 (50%)
Gastric cancer *N* = 10	9/35 (25.7%)
Appendicealmucinous carcinoma *N* = 55	9/55 (16.4%)
*N* = 143	81/143 (56.6%)

**Table 4:** PpIX contents in peritoneal metastases according to the primary site [12,19]. N = number of patients.

Primary Sites	PpIX Content in Peritoneal Metastasis
Ovarian cancer *N* = 10	0.0185 ± 0.0017
Mesothelioma *N* = 5	0.0156 ± 0.0105
Pancreas cancer *N* = 4	0.0104 ± 0.0108
Colorectal cancer *N* = 29	0.0107 ± 0.0009
Gastric cancer *N* = 10	0.0016 ± 0.0017
Appendicealmucinous carcinoma *N* = 15	0.0025 ± 0.0016

**Table 5:** Results of ALA PDD clinical trials for peritoneal metastasis. N = number of patients.

Authors	Disease	Administration Rout	Dose (mg/kg)	Incubation Time (h)	Sensitivity	False Positive	Specificity
Loning M. [20]	ovarian cancer (*N* = 29)	intraperitoneal	30	5	92%	2%	
Liu Y. [8]	ovarian cancer (*N* = 20)	oral	20	2	95%	0%	100%
Yonemura Y. [12]	peritoneal metastasis (*N* = 138)	oral	20	2	46%	0%	100%
Murayama Y. [29]	gastric cancer (*N* = 13)	oral	10–15	3	100%	0%	100%
Hillemanns P. [26]	ovarian cancer (*N* = 26)	oral	10	9–16	75%	0%	100%
